# Does a whole millet grain-based diet replace whole corn grain in non-forage diets for goat kids?

**DOI:** 10.1007/s11250-026-05165-9

**Published:** 2026-06-22

**Authors:** Francisca Leila Araujo dos Santos, Daniel Louçana da Costa Araújo, Luana Michele Pereira Gonçalves, Maria Clara Silva Soares, Fabiano Alves Lopes, Dayane Francisca Higino Miranda, Arnaud Azevêdo Alves, Henrique Nunes Parente, Marcos Jacome de Araújo, Francisco Naysson de Sousa Santos, Rafael Silvio Bonilha Pinheiro, Michelle de Oliveira Maia Parente

**Affiliations:** 1 Post-Graduation Program in Tropical Animal Science, Universidade Federal do Piauí, Teresina, PI 64049-550 Brazil; 2 Department of Animal Science, Universidade Federal do Piauí, Teresina, PI 64049-550 Brazil; 3 Post Graduation Program in Animal Science, Universidade Federal do Maranhão, Chapadinha, MA 65500-000 Brazil; 4 Department of Animal Science, Universidade Federal do Piauí, Bom Jesus, PI 64900-000 Brazil; 5 Department of Animal Science, Universidade Estadual Paulista “Júlio de Mesquita”, Botucatu, SP 18618-681 Brazil

**Keywords:** Average daily gain, Carcass, *Pennisetum glaucum*, Rumination time

## Abstract

Twenty-one Anglo-Nubian goat kids (21.6 ± 2.9 kg) were assigned to a completely randomized design to evaluate the effects of different whole grain-based diets on performance, ingestive behavior, physiological parameters, and carcass characteristics. The experimental diets consisted of a control diet (CON), containing 10% hay and 90% concentrate, and two non-forage diets: a whole corn grain-based diet (WC) and a whole millet grain-based diet (WM), both composed of 20% commercial pellet and 80% the respective whole grain. The non-forage diets reduced dry matter intake (*P* = 0.002); however, crude protein intake was reduced only in the WC diet (*P* = 0.001). The CON diet increased fiber intake (*P* < 0.001) and resulted in the longest rumination time (*P* = 0.026), whereas the WM diet showed the highest ether extract (EE) intake (*P* < 0.001). The WC diet altered the feed-sorting behavior of the goat kids and consequently showed the highest DM digestibility (*P* = 0.042). The WC diet showed higher EE digestibility than the CON diet (*P* = 0.025), whereas EE digestibility in the WM diet did not differ from that in the other diets. However, the WC diet reduced average daily gain (*P =* 0.006), and slaughter weight (*P =* 0.025), without affecting carcass weight, carcass yield, or leg tissue composition (*P >* 0.05). Physiological parameters were not influenced by diet (*P* > 0.05). Overall, WM can replace WC in non-forage diets, maintaining satisfactory performance, carcass characteristics, and leg tissue composition in goat kids.

## Introduction

Since the 1970s, whole grain–based diets (WGD) without forage have been investigated in the United States, raising discussion about their applicability in beef cattle feedlots (Paulino et al. 2013; Contadini 2017). The adoption of this feeding strategy is justified when the objective is to achieve high weight gain rates, improve feed efficiency, and simplify daily feed management (Contadini 2017).

In feedlot production systems, corn is the primary energy source used in animal diets and serves as the main ingredient in WGD (Fabino Neto et al. 2022). This feeding strategy, also referred to as a non-forage diet, typically consists of 15–20% commercial pellets and 80–85% whole corn grain (WC) (Paulino et al. 2013). Despite its potential advantages, this approach is not widely adopted in ruminant nutrition, not necessarily because of economic constraints, but rather because of resistance or apprehension among many nutritionists.

Although ruminants have evolved to consume forage-based diets, non-forage diets may represent a viable technological alternative in situations where grains are economically accessible, and the supply or management of roughage is limited. Moreover, increasing concern regarding the environmental impact of livestock production has highlighted the role of dietary composition in modulating enteric methane emissions. The fermentation of highly fermentable carbohydrates, such as the starch present in corn and millet, promotes greater propionate production in the rumen, thereby reducing the availability of hydrogen for methanogenesis. This shift in ruminal fermentation pathways may contribute to the mitigation of enteric methane emissions. Thus, the partial or total replacement of forage with grain may represent a feasible strategy to reduce the environmental footprint of animal production without compromising animal performance (Sun et al. 2022). Recently, several studies have evaluated non-forage diets for finishing lambs (Mendes et al. 2018; Carlis et al. 2021); however, information regarding goat kids remains scarce (Bilal et al. 2025).

The extensive use of corn grain in non-forage diets increases feeding costs because corn is also highly demanded for both animal feed and human consumption. Therefore, the search for alternative ingredients is necessary, and millet (*Pennisetum glaucum*) grain has emerged as a promising option because of its favorable nutritional composition, including starch (63.2%), crude protein (CP) (13.6%), ether extract (EE) (7.8%), crude fiber (2.8%), ash (2.1%), and dry matter (DM) content (92.5%) (Sharma et al. 2016). Moreover, millet is considered an advantageous crop for feeding purposes because of its nutritional superiority over several other cereal grains (Jenipher et al. 2024). Compared with corn, millet contains higher CP but slightly lower starch content, whereas corn contains approximately 9% CP and 70% starch (Timm et al. 2020).

According to the same authors, corn starch granules are closely associated with proteins, particularly in the grain endosperm. In Brazil, flint-type corn, characterized by a hard endosperm, predominates (Carlis et al. 2023), which may limit the ruminal bacterial access to nutrients. We hypothesized that animals fed a whole millet (WM) diet would exhibit increased nutrient digestibility, average daily gain (ADG), and improved carcass characteristics, without changes in feed sorting behavior. Thus, the aim of this study was to evaluate the replacement of WC with WM in non-forage diets for feedlot- finished goat kids, focusing on performance, ingestive behavior, physiological parameters, and carcass characteristics.

## Materials and methods

### Location, animals, and experimental facilities

The experiment was conducted at the Animal Science Department, Center of Agricultural Sciences, Federal University of Piauí (5°02′30″S; 42°47′00″W; altitude: 69.37 m), located in Teresina, Piauí, Brazil. This study was approved by the Ethics Committee on Animal Use under protocol number 733/2022.

Twenty-one Anglo-Nubian goat kids, with an initial body weight (BW) of 21.6 ± 2.9 kg and aged 150 ± 8 days, were individually housed in covered pens (1.0 × 1.3 m) with concrete floors and metal railings, equipped with a drinker, feeder, and mineral salt trough. The experimental period lasted 59 days, consisting of 19 days for adaptation to the diets and management conditions, followed by a 40-day finishing period. On the first day of the adaptation period, the animals were dewormed (levamisole, 2 mL/animal) and vaccinated against clostridiosis (Excell 10^®^, 2 mL/animal).

### Experimental design, treatments, and management

The experiment followed a completely randomized design with three treatments and seven replicates. The chemical composition of the ingredients is presented in Table 1. The control (CON) diet was formulated according to NRC (2007) recommendations (Table 2) to meet the requirements of growing goat kids (20 kg BW and an expected ADG of 150 g/d) and consisted of 90% concentrate and 10% Tifton-85 hay. The non-forage diets were formulated according to the manufacturer’s recommendations and included 20% commercial protein pellet and 80% of either WC or WM.

**Table 1:** Chemical and physical composition of the ingredients of the experimental ingredients (% Dry Matter)

Nutrients^a^, %	Ingredients				
	Whole corn	Tifton 85 Hay	Whole Millet	Pellet^b^	Concentrate^c^
Dry matter	84.5	86.5	80.7	81.3	68.8
Crude protein	7.7	6.7	15.5	36.9	16.6
Ether Extract	4.1	1.0	5.9	5.5	2.6
Ash	1.4	4.3	1.5	11.8	2.7
aNDFom	17.1	80.9	25.1	28.9	20.3
Total-CHO	86.7	87.9	77.1	45.8	69.6
NF-CHO	69.6	7.0	52.0	16.9	57.4
Particle size (% of DM)					
Screen, 19 mm	0	0	0	0	0
Screen, 8 mm	81.7	32.9	0	7.3	0
Screen, 4 mm	18.3	20.2	1.9	92.7	18.2
Screen,1.18 mm	0	45.7	98.1	0	31.8
Bottom pan,<1.18 mm	0	1.1	0	0	50.0

^a^ aNDFom: Neutral detergent fiber assayed with a heat stable amylase and expressed as corrected for ash;

^b^ Ingredients: Soybean meal, wheat bran, corn grain, urea, Dicalcium phosphate, Calcitic limestone, Sodium chloride (common salt), Livestock sulfur, Iron sulfate, Copper sulfate, Manganese sulfate, Zinc oxide, Cobalt sulfate, Calcium iodate, Sodium selenite, Vitamin A, Vitamin B3, Vitamin E, Propionic acid, Formic acid, Ammonium propionate, B.H.A., Ethoxyquinyl, Citric acid, and Phosphoric acid

^c^ Concentrate from a control diet

**Table 2:** Proportion of ingredients, physical characterization and chemical composition of experimental diets

Item	Diets^d^		
	Control	WC	WM
Ingredients, % DM			
Ground corn	54.5		
Soybean meal	16.0		
Wheat bran	17.1		
Tifton-85 hay	10.0		
Whole corn grain		80.0	
Whole Millet grain			80.0
* Pellet* ^*a*^		20.0	20.0
Limestone	0.2		
Ammonia Chloride	0.4		
Sodium bicarbonate	0.8		
Mineral salt^b^	1.0		
Chemical composition, % DM^c^			
Dry matter	83.0	83.8	80.7
Crude protein	15.7	13.6	19.7
Ether extract	2.6	4.4	5.8
Ash	3.2	3.5	3.6
aNDFom	28.2	19.4	25.8
Total carbohydrates	78.4	78.5	70.9
Non-fibrous carbohydrates	58.1	59.0	45.0
ME, Mcal/kg DM	2.7	2.9	3.0
Particle size, % DM			
Screen, 19 mm	0	0	0
Screen, 8 mm	3.3	66.8	1.5
Screen, 4 mm	18.4	33.2	20.0
Screen, 1.18 mm	33.2	0	78.5
Bottom pan, < 1.18 mm	45.1	0	0

^a^ Ingredients: Soybean meal, wheat bran, corn grain, urea, Dicalcium phosphate, Calcitic limestone, Sodium chloride (common salt), Livestock sulfur, Iron sulfate, Copper sulfate, Manganese sulfate, Zinc oxide, Cobalt sulfate, Calcium iodate, Sodium selenite, Vitamin A, Vitamin B3, Vitamin E, Propionic acid, Formic acid, Ammonium propionate, B.H.A., Ethoxyquinyl, Citric acid, and Phosphoric acid;

^b^ Guarantee levels per kilogram of the product according to the manufacturer: Sodium (min.) 147 g; Calcium (min.) 120 g; Phosphorus (min.) 87 g; Sulfur (min.) 18 g; Zinc (min.) 3800 mg; Iron (min.) 1800 mg; Manganese (min.) 1.300 mg; Fluorine (max.) 870 mg; Copper (min.) 590 mg; Molibdˆenio (Mo) 300 mg; Iodine (min.) 80 mg; Cobalt (min.) 40 mg; Chromium (min.) 20 mg; Selenium (min.) 15 mg. Phosphorus (P) solubility in 2% citric acid (min): 95%

^c^ aNDFom: Neutral detergent fiber assayed with a heat-stable amylase and expressed as corrected for ash; ME: Metabolizable energy

^d^ WM: non-forage diets with whole millet grain; WC: non-forage diets with whole corn grain

During the first 19 days, the animals were subjected to the adaptation protocol recommended by the pellet feed manufacturer, as follows: days 1–3, total mixed ration (TMR) equivalent to 1.5% BW; days 4–7, TMR equivalent to 2% BW; days 8–11, TMR equivalent to 3% BW; days 12–15, TMR equivalent to 3.5% fasting BW; and days 16–19, roughage was completely withdrawn, and the animals received TMR equivalent to 3.5% to 4% fasting BW.

The experimental diets were supplied twice daily, at 8:00 a.m. and 4:00 p.m., and orts were collected and weighed daily to determine DM intake (DMI). Daily feed supply was adjusted to allow approximately 10% orts, depending on intake. Water and mineral salt were available *ad libitum* throughout the experimental period. Orts from the offered feed were collected twice weekly, identified, and stored frozen for subsequent determination of nutrient intake. At the end of the experimental period, samples were thawed and pooled by animal.

The previously formulated CON concentrate and hay were weighed separately using an electronic scale and manually mixed in each feeder before feeding. The high-grain diets were prepared similarly, with the commercial pellet and WC or WM weighed separately, manually mixed immediately before feeding, and then offered to each animal.

The animals could not be subjected to prolonged fasting because of management constraints; therefore, the goat kids were weighed on 3 consecutive days at both the beginning and the end of the experimental period. ADG was calculated as the difference between final BW and initial BW divided by the number of experimental days.

At the end of the feedlot period, the animals remained in the pens for an additional 3 days for total feces collection using nylon bags, as described by Santos et al. (2022). Nutrient digestibility coefficients were calculated using the following equation: Digestibility coefficient = [(kg of ingested nutrient − kg of excreted nutrient)/(kg of ingested nutrient)] × 100.

### Sample collection and chemical analyses

At the end of the feedlot period, samples of the experimental diets and orts were thawed and pooled by animals. The samples were ground through a 1-mm screen using a Wiley mill (Marconi, Piracicaba, SP, Brazil). DM (Method 934.01), ash (Method 942.05), EE (Method 954.05), and total nitrogen (Method 968.06) were determined according to AOAC (1990). CP was calculated by multiplying total nitrogen by 6.25.

Neutral detergent fiber (NDF), assayed with heat-stable amylase and expressed exclusive of ash, was determined according to Mertens et al. (2002). Total carbohydrates and non-fiber carbohydrates were determined according to Sniffen et al. (1992) and Mertens et al. (2002), respectively.

The mean particle size of the diets was determined using the Penn State Particle Size Separator method (Lammers et al. 1996). Particle size distribution was assessed according to Kononoff et al. (2003), based on the proportions of the sample retained on sieves with mesh sizes of 19, 8, 4, and 1.18 mm (Table 2).

Dietary metabolizable energy (ME) content was estimated from nutrient digestibility data, assuming that 1 kg of total digestible nutrients corresponds to 4.409 Mcal of digestible energy and that 1 Mcal of digestible energy equals 0.82 Mcal of ME (NRC, 2007).

### Ingestive behavior, physiological parameters, and water intake

Individual observations of the animals were conducted on the 31st day of the experimental period to record the following activities: feeding, ruminating, idling, and other activities. Ingestive behavior was evaluated at 5-min intervals over a continuous 24-h period, beginning at 8:00 a.m., according to the method described by Johnson and Combs (1991).

Data were recorded by trained observers (one per seven goat kids) positioned to minimize interference with animal behavior. Observer shifts were changed every 6 h, and artificial lighting was used, during the night period, following the same procedures adopted at the beginning of the experiment.

Feeding and rumination efficiencies of DM and NDF were calculated based on the time spent feeding and ruminating (min/day) and the respective daily intake of DM and NDF (Azevedo et al. 2013). Intake rate and rumination efficiency for DM and NDF were expressed as the ratio between feeding or rumination time and the amount of nutrient (DM or NDF) consumed per day.

Physiological responses were evaluated at 06:00 a.m., 10:00 a.m., 2:00 p.m., and 6:00 p.m. over 7 consecutive days (days 32–38 of the experimental period). The following parameters were measured: rectal temperature (RT), body temperature (BT), heart rate (HR), and respiratory rate (RR). On these days and at the same time points, environmental variables (air temperature and relative humidity) were also recorded.

RT was measured using a veterinary clinical thermometer (Highmed, Termo KT-DT 4B, Belo Horizonte, MG, Brazil), inserted into the rectum for 2 min, and recorded in °C. BT was measured using a laser thermometer (Akrom, model KR380, Porto Alegre, RS, Brazil) and calculated as the arithmetic mean of the temperatures recorded at the temple, base of the tail, and lateral body surface (Machado et al. 2019). RR was determined by direct observation of left flank movements, as described by Kawabata et al. (2013), by counting flank movements for 15 s and multiplying by four to obtain breaths per minute. HR was measured using a veterinary stethoscope positioned over the left thoracic region, and the number of beats per minute was recorded (Diffay et al. 2004).

For water intake measurements, the volume of water offered and refused was weighed using 10-L plastic buckets. Buckets were weighed before water delivery and again after 24 h to determine water intake via drinking. Water was supplied daily at 7:30 a.m. Two additional buckets containing water were placed in the shed to estimate daily evaporation losses. Water intake via feed and total water intake were calculated according to Silva et al. (2024).

### Slaughter procedures and carcass characteristics

At the end of the 40-day feeding trial, the animals were fasted for 14 h with free access to water and subsequently weighed to determine BW at slaughter. Hot carcass weight was recorded immediately after slaughter. All procedures were performed at a commercial abattoir in accordance with the regulations established by the Brazilian Regulation of Industrial and Sanitary Inspection of Products of Animal Origin (Brasil 2017).

After slaughter, the carcasses were suspended by the calcaneal tendon and chilled at 4 °C for 24 h. Subjective carcass evaluations were performed for finishing (scored from 1 = very lean to 5 = very fat), conformation (1 = poor to 5 = excellent), and renal fat deposition (1 = low to 3 = high), according to Cezar and Sousa (2007). The kidneys and perirenal fat were then removed, and their weights were deducted from the carcass weight.

The *longissimus thoracis* (LT) muscle was exposed by a transverse cut between the 12th and 13th ribs. Subcutaneous fat thickness (SFT) was measured on both sides of the carcass using a digital caliper (DIGIMESS, São Paulo, SP, Brazil). The exposed LT area was determined using a plastic grid. Mean values for SFT and LT area were calculated from measurements obtained on the right and left sides of each carcass.

### Statistical analysis

Data were analyzed using analysis of variance with the MIXED procedure of SAS, considering the experimental diet as a fixed effect. When significant effects were detected (*P* < 0.05), means were compared using Tukey’s test. Initial BW was included in the model as a covariate. Residual normality was assessed using the Shapiro-Wilk test.

Physiological parameters were analyzed as repeated measures over time. The most appropriate covariance structure was selected based on the corrected Akaike Information Criterion (AICC) and Bayesian Information Criterion (BIC), and the model with the lowest AICC or BIC value was considered the best fit. The selected covariance structures were first-order autoregressive [AR (1)] for HR and BT, and compound symmetry (CS) for RT and RR.

## Results

### Intake, growth, and nutrient utilization

Non-forage diets reduced DM intake, expressed in g/day (*P* = 0.002) and as % BW (*P* = 0.001), which consequently decreased organic matter intake (*P* = 0.001) and total carbohydrates intake (*P* = 0.004) compared with the CON diet (Table 3). However, non-fiber carbohydrates intake was not affected by diet (*P* = 0.274), averaging 287 ± 68 g/day (Table 3).

**Table 3:** Effects of replacement of whole corn grain by whole millet grain on nutrient intake, digestibility coefficient and growth performance of goat kids

Item^a^	Diets^a^			SEM^b^	*P*-value^c^
	Control	WC	WM		
Intake					
Dry Matter, g/d	681ª	494^b^	560^b^	30.554	0.002
Dry Matter, %BW	2.76^a^	2.10^b^	2.28^b^	0.084	0.001
Ash, g/d	18.18	17.47	15.57	1.205	0.689
Organic matter	663.0^a^	476.7^b^	544.9^b^	29.837	0.001
Crude protein, g/d	103ª	61^b^	98^a^	5.997	0.001
Neutral detergente fiber, g/d	205^a^	99^c^	147^b^	11.544	< 0.001
Ether Extract, g/d	20.9^b^	22.7^b^	36.7^a^	2.053	< 0.001
Total carbohydrates, g/d	525^a^	382^b^	411^b^	23.883	0.004
Non-fibrous carbohydrates, g/d	318	280	268	15.221	0.274
Metabolizable energy, Mcal/d	1.84^a^	1.38^b^	1.69^ab^	0.087	0.012
Digestibility, %					
Dry Matter	86.15^b^	91.44^a^	86.69^b^	0.866	0.042
Organic matter	87.62	90.27	88.07	0.655	0.282
Crude protein	85.97	81.94	86.62	0.932	0.142
Neutral detergente fiber	77.16	76.97	78.02	0.886	0.804
Ether Extract	87.81^b^	90.74^a^	88.53^ab^	0.424	0.025
Total carbohydrates	87.93	91.48	88.29	0.744	0.149
Non-fibrous carbohydrates	92.67	98.88	94.70	7.729	0.470
Growth					
Initial weight, kg	21.5	21.3	22.2	-	-
Final weight, kg	27.7^a^	24.9^b^	27.3^a^	1.001	0.006
Average daily gain, g/d	149.2^a^	79.1^b^	138.5^a^	12.045	0.006
Feed: Gain ratio	0.21^a^	0.16^b^	0.24^a^	0.013	0.008

^a^ WM: non-forage diets with whole millet grain; WC: non-forage diets with whole corn grain;

^b^ SEM: Standard Error of Mean;

^c^ Values with different superscripts are different for *P* < 0.05

Because of differences in EE content among diets, animals fed the WM diet exhibited the highest EE intake (*P* < 0.001). An effect was also observed for NDF intake, which was higher (*P* < 0.001) in goat kids fed the CON diet, followed by those fed WM and WC (Table 3). The higher fiber intake in the CON diet was associated with increased water intake via drinking (g/day; *P* = 0.043) and a tendency toward greater total water intake (*P* = 0.056) compared with WC, whereas WM did not differ from the other diets (Table 4). Water intake via feed (*P* = 0.291) and total water intake per kilogram of DM consumed (*P* = 0.433) were not affected by diet.

**Table 4:** Physiological parameters, feeding behavior, and water intake of goat kids fed non-forage diets containing whole millet grain as a replacement for whole corn grain

Item^a^	Diets^b^			SEM^c^	Effect^d^		
	Control	WC	WM		Diet	Hour	D×H
Physiological parameters							
HR, beats/min	100.14	100.92	99.98	0.913	0.904	0.026	0.633
RR, mov/min	32.14	33.68	32.94	0.517	0.413	< 0.001	0.836
RT, °C	38.88	38.86	38.92	0.036	0.746	0.048	0.843
BT, °C	36.23	36.20	36.27	0.024	0.185	< 0.001	0.844
Feeding behavior							
Feeding, min/d	269.59	237.28	173.79	17.738	0.091		
Ruminating, min/d	135.39^a^	63.14^b^	65.08^b^	13.332	0.026		
Iddle, min/d	1023^b^	1132^ab^	1186^a^	23.423	0.009		
OA, min, d	26.34	22.20	26.34	3.490	0.696		
Feeder visits, number	53.91	47.45	34.76	3.547	0.091		
Feed efficiency							
Dry Matter, g/h	134.33	170.75	206.57	15.300	0.174		
NDF, g/h	32.52	50.63	50.63	5.267	0.237		
Rumination efficiency							
Dry Matter, g/h	315.90^b^	704.49^a^	632.08^a^	66.538	0.005		
NDF, g/h	75.31^b^	200.94^a^	163.86^a^	19.900	0.007		
Water intake							
WID, g/d	3158^a^	1745^b^	2174^ab^	255.09	0.043		
WIF, g/d	331	230	299	28.69	0.291		
TWI, g/d	3489	1975	2473	282.25	0.056		
WI.DMI	5049	3960	4300	326.38	0.433		

^a^ HR: Heart rate; RR: Respiratory rate; RT: rectal temperature; BT: body temperature; OA: other activities; NDF: neutral detergent fiber; WID: water intake via drinking fountain; WIF: water intake via feed; TWI: total water intake; WI.DMI: Total water intake per kg of dry matter ingested;

^b^ WM: non-forage diets with whole millet grain; WC: non-forage diets with whole corn grain;

^c^ SEM: Standard Error of Mean;

^d^ D × H: Interaction between diet and hour; Values with different superscripts are different for *P* < 0.05

The WC diet reduced CP intake (*P* = 0.001) and decreased ME intake (*P* = 0.012) compared with the CON diet, whereas ME intake in animals fed WM did not differ from that of the other diets (Table 3). As a result, animals fed WC showed the lowest ADG (*P* = 0.006) and feed: gain ratio (*P* = 0.008). Higher ADG values were observed in animals fed CON and WM (149.2 g/d and 138.5 g/d, respectively), which did not differ from each other.

Regarding nutrient digestibility (Table 3), the WC diet resulted in the highest DM digestibility (*P* = 0.042). The WC diet also showed the highest EE digestibility, differing significantly from the CON diet (*P* = 0.025). By contrast, EE digestibility in the WM diet was intermediate and did not differ from that of the other diets (*P* > 0.05).

### Ingestive behavior and physiological parameters

**Fig. 1:**
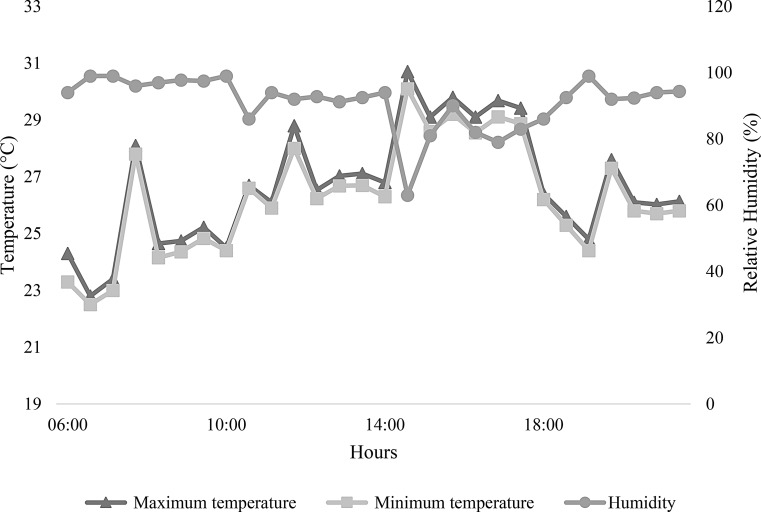
Average values of maximum and minimum air temperatures (AT), and relative humidity (RH) obtained during physiological measures

**Fig. 2:**
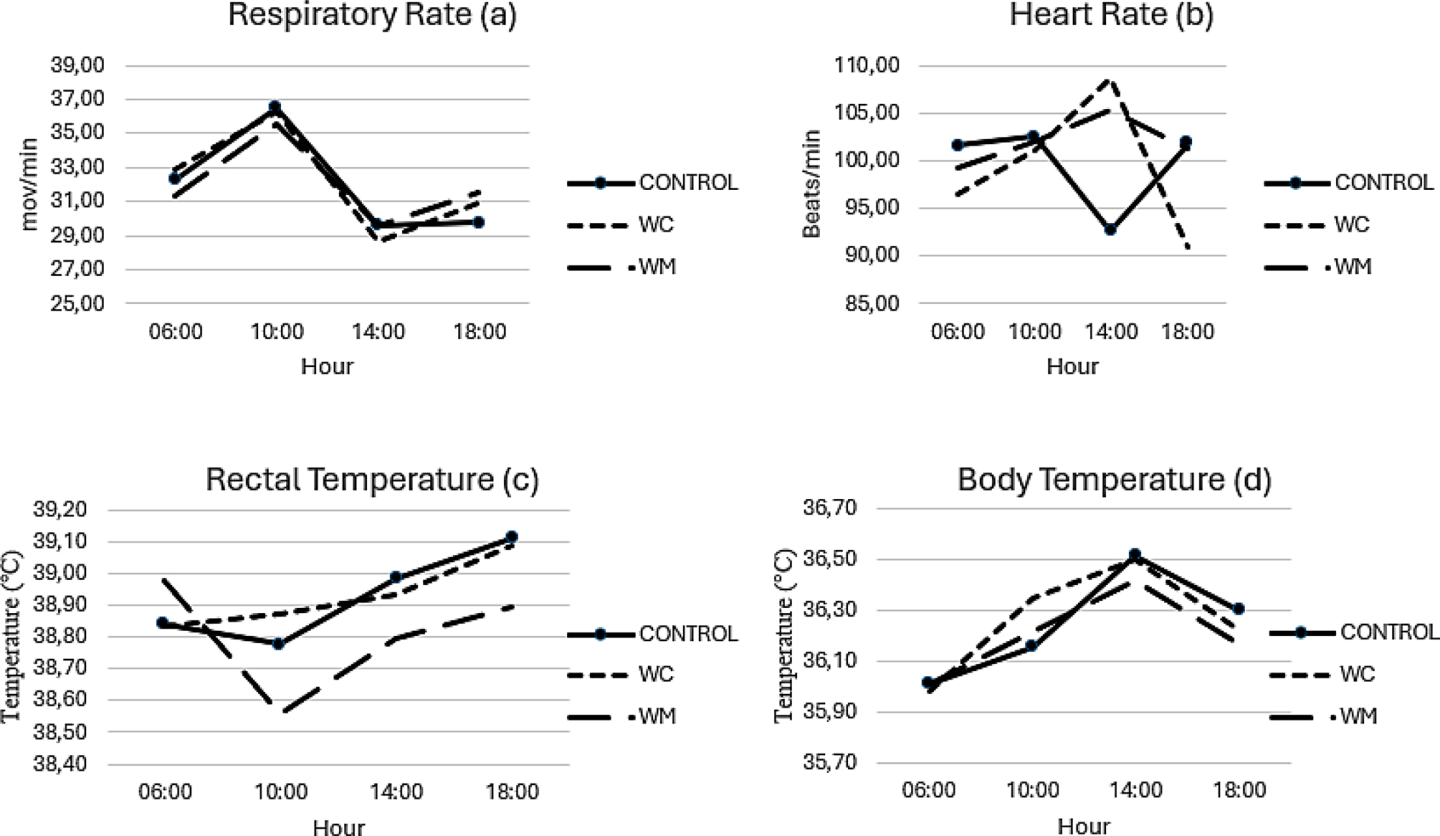
Effect of collection hour on physiological parameters of goat kids fed non-forage diets containing whole millet grain as a replacement for whole corn grain

The experimental diets did not affect RR (*P* = 0.413), HR (*P* = 0.904), BT (*P* = 0.185), or RT (*P* = 0.746) (Table 4). No diet × hour interaction was detected (*P* > 0.05); however, a significant effect of hour was observed (*P* < 0.05) for all physiological variables, with increasing mean values associated with higher ambient temperatures throughout the day (Figs. 1 and 2). Regarding ingestive behavior, the WM diet tended to reduce feeding time and the number of feeder visits (*P* = 0.091), whereas animals fed the CON diet spent more time ruminating (*P* = 0.026). The WM diet increased idle time (*P* = 0.009) compared with the CON diet but did not differ from the WC diet. Feed efficiency for DM (*P* = 0.174) and NDF (*P* = 0.237) was not affected by diet. However, the CON diet reduced rumination efficiency for DM (*P* = 0.005) and NDF (*P* = 0.007) compared with the non-forage diets (Table 4).

### Carcass characteristics and leg tissue composition

The WC diet reduced slaughter weight (*P* = 0.025); however, no effects were observed on hot carcass weight (*P* = 0.665), hot carcass yield (*P* = 0.296), carcass conformation (*P* = 0.542), or SFT (*P* = 0.879). The CON diet decreased both the kidney fat score (*P* = 0.004) and kidney weight (*P* = 0.018) compared with the non-forage diets (Table 5). Additionally, WC tended to reduce the finishing score (*P* = 0.061), whereas WM tended to increase the LT area (*P* = 0.054).

**Table 5:** Weight at slaughter and carcass characteristics of goat kids fed non-forage diets containing whole millet grain as a replacement for whole corn grain

Item^a^	Diets^b^			SEM^c^	*P*-value^d^
	Control	WC	WM		
Weight at slaughter, kg	27.6^a^	25.2^b^	27.7^a^	1.048	0.025
Hot carcass weight, kg	13.30	12.67	12.79	0.505	0.665
Hot carcass yield, %	48.0	50.4	46.2	1.081	0.296
Kidney fat score	2.34^b^	3.02^a^	2.82^a^	0.099	0.004
Kidney fat, g	524^b^	648^a^	681^a^	45.44	0.018
SFT, mm	1.06	0.99	1.04	0.048	0.879
LT area, cm^2^	14.21	14.95	17.19	0.639	0.054
Finishing	2.19	1.48	1.63	0.160	0.061
Conformation	2.19	1.91	2.19	0.160	0.542

^a^ SFT: Subcutaneous fat thickness; LT: Longissimus thoracis muscle area

^b^ WM: non-forage diets with whole millet grain; WC: non-forage diets with whole corn grain;

^c^ SEM: Standard Error of Mean;

^d^ Values with different superscripts are different for *P* < 0.05

Regarding leg tissue composition (Table 6), no differences were detected (*P* > 0.05) in the weight or proportion of muscle, fat, bone, or other tissues. Likewise, the muscle: fat (*P* = 0.383) and muscle: bone ratios (*P* = 0.578) were not affected by diet.

**Table 6:** Leg tissue composition of goat kids fed non-forage diets containing whole millet grain as a replacement for whole corn grain

Item	Diets^a^			SEM^b^	*P*-value^c^
	Control	WC	WM		
Leg weight, kg	1.87	1.56	1.77	0.109	0.232
Muscle					
Kg	1.23	1.14	1.13	0.047	0.250
%	66.25	65.50	63.38	0.834	0.384
Fat					
kg	0.11	0.12	0.15	0.009	0.357
%	6.57	7.25	8.28	0.515	0.401
Bone					
kg	0.39	0.35	0.37	0.013	0.204
%	21.41	20.47	21.22	0.442	0.651
Other tissues					
kg	0.08	0.07	0.10	0.007	0.337
%	3.93	4.86	5.63	0.335	0.109
Ratios					
Muscle: Fat	11.87	11.46	7.92	1.275	0.383
Muscle: Bone	3.13	3.26	3.03	0.091	0.578

^a^ WM: non-forage diets with whole millet grain; WC: non-forage diets with whole corn grain;

^b^ SEM: Standard Error of Mean;

^c^ Values with different superscripts are different for *P* < 0.05

## Discussion

Corn grain is considered the primary energy source in diets for ruminants finished in feedlot systems (Carlis et al. 2021; Pinto and Millen 2018). However, in Brazil, flint-type endosperm corn predominates (Carlis et al. 2023), which reduces the accessibility of starch granules to ruminal microorganisms, particularly when supplied as whole grain (Owens et al. 1986; McAllister et al. 1994). This characteristic, combined with the lower DM and EE intake observed in animals fed the WC diet may explain the reduction in ME intake compared with the CON diet. 

The reduction in DMI in animals fed the WC diet, together with its lower CP concentration, contributed to reduced protein intake. Although WM also reduced DMI, this negative effect on protein intake was not observed, likely because its higher dietary protein content compensated for the lower intake. Consequently, only goats receiving the WC diet showed reductions in ADG and BW at slaughter. Nevertheless, the results indicate that the total replacement of WC with WM in non-forage diets maintained ADG similar to that observed in the control group.

The average DMI of goat kids fed the CON diet was close to the value recommended by the NRC (2007) for 8-month-old animals weighing 20 kg BW (700 g/day). Although no significant differences in ADG were observed between the CON and WM diets, WM resulted in an ADG approximately 7% lower than the NRC recommendation (150 g/day) for animals of similar age and BW. In contrast, animals fed the WC diet showed an approximately 47% reduction relative to the recommended value.

In ruminants, intake under high-concentrate diets is regulated primarily by the animal’s energy requirements and metabolic feedback mechanisms (Allen 1996). This explains the absence of differences in ADG between animals fed WM and those fed the CON diet, despite the lower DMI observed with WM. Because non-forage diets had greater energy density than the CON diet (Table 2), a reduction in DMI accompanied by similar ADG was expected. However, this pattern was not observed in animals fed the WC diet.

Energy intake greatly affects feed efficiency and the overall performance of small ruminants (Mahgoub et al. 2025). Nevertheless, in growing goats, reduced protein intake may explain decreases in ADG at younger ages (Lu and Potchoiba 1990). As observed in this study, the WC diet not only reduced energy intake but also led to an approximately 40% reduction in protein intake, which likely contributed to the lower ADG.

Goats are known for selective feeding behavior (Lopes et al. 2023). Differences in particle size distribution and digestible feed fractions can alter intake patterns, thereby positively or negatively affecting DM intake and animal performance (Gomes et al. 2020; Malik et al. 2020), as well as nutrient utilization (Malik et al. 2023).

Goat kids fed the WM diet tended to spend less time feeding, which is consistent with their lower DMI. However, the reduction in DMI observed in animals fed the WC diet was not accompanied by a proportional reduction in feeding time, suggesting the occurrence of selective feeding behavior, a well-known characteristic of goats. Feeding time in animals fed WC and WM was 12% and 35% lower, respectively, than that in animals fed the CON diet, which may be explained by differences in particle size distribution.

As shown in Table 2, approximately 78% of particles in the WM and CON diets were retained on the 1.18-mm sieve, whereas WC particles were predominantly retained on sieves ≥ 4 mm. As noted above, goats are highly selective animals that are adaptable to diverse feeding conditions, and larger particle sizes may further facilitate feed selection, as reported by Ackermans et al. (2019). Nevertheless, a recent study reported that goats are able to select feed particles ground to pass through 3-mm sieves (Malik et al. 2025).

In this study, feed sorting behavior in animals fed the WC diet may have led to preferential consumption of pelleted particles, which are more readily fermentable, as reported by Animut and Goetsch (2008) and Morand-Fehr (2003). Another factor influencing feed selection and intake is particle hardness (Ackermans et al. 2019). According to these authors, goats tend to select less hard particles, possibly to avoid excessive tooth wear.

There are few data regarding the feeding of goats with high-concentrate or non-forage diets. In feedlot systems, goats tend to select fiber particles in high-concentrate diets, preferring coarser feeds over fine concentrate particles, unlike cattle and sheep, probably because of a physiological response aimed at maintaining ruminal pH (Malik et al. 2025). Regardless of inherent particle-size preference, goats can adapt their feeding behavior according to environmental conditions and management constraints (Melado 2016; Morand-Fehr 2003). Gomes et al. (2020) reported that feed-restricted goats modified their natural behavior by increasing their preference for smaller particles, typically concentrates, which represent the most energy-dense fraction of the diet. Because of this selective behavior, goats preferentially consume more digestible feed fractions (Animut and Goetsch 2008).

Pelleting increases the feed surface area, and may consequently improve digestibility (Bateman et al. 2009). Accordingly, the WC diet resulted in the greatest DM digestibility and higher EE digestibility than the CON diet, whereas the WM diet did not differ from the other diets. Pellets may facilitate faster and more efficient microbial degradation than coarse particles such as WC (Allen and Mertens 1988).

In addition to feed selection, WC may have remained in the rumen for a longer period, contributing to reduced DMI, as suggested by Bilal et al. (2025). Prolonged ruminal may reduce passage rate and extend the action of microbial digestive enzymes (Allen and Mertens 1988). Therefore, the slight increase in DM digestibility observed in goat kids fed the WC diet was insufficient to promote greater weight gain.

According to Van Soest (1994), non-forage diets based in whole grains may increase rumination time and saliva production, thereby contributing to ruminal pH stabilization and fiber digestion. However, in the present study, non-forage diets reduced rumination time, because of lower DMI, without impairing NDF digestibility. Despite the reduction in rumination time, rumination efficiency for both DM and NDF increased compared with the CON diet. Because these variables are expressed as a function of rumination time relative to nutrient intake, the combination of reduced rumination time and lower NDF intake in non-forage diets likely explains the observed increase in rumination efficiency.

High-concentrate diets with very low physically effective NDF may increase the risk of ruminal acidosis (Carlis et al. 2021; Chen et al. 2021). Nevertheless, recent studies with small ruminants, particularly lambs (Carlis et al. 2021; Eckermann et al. 2026), have reported that WC-based diets maintained satisfactory finishing performance without clinical signs of ruminal acidosis. To our knowledge, few studies have evaluated non-forage diets in goats. However, Bilal et al. (2025), when comparing different physical forms of diets, reported higher ruminal pH, whereas DMI and ADG were reduced in goats fed a texturized diet, consisting of WC combined with ground and pelleted ingredients.

In the present study, the WM diet maintained energy supply balance while potentially reducing the risks associated with excessive intake of rapidly fermentable carbohydrates (Allen 1996) compared with the CON diet. This response was not observed in animals fed the WC diet, in which changes in feed sorting behavior combined with reduced intake resulted in lower ADG, despite the greater DM digestibility. Feeding the WM diet resulted in 42% greater weight gain than feeding the WC diet, demonstrating that WM is a viable alternative to WC in grain-based diets. Thus, our hypothesis that WM would increase nutrient digestibility was not supported by the present data. However, the WM diet maintained ADG similar to that observed in animals fed the CON diet and resulted in better performance than the WC diet.

The absence of differences in RR, HR, BT, and RT among diets indicates that the treatments did not impose metabolic or thermal stress on the animals. Under heat stress, animals typically increase RR and RT to enhance heat dissipation; however, when diets are nutritionally balanced and do not induce severe digestive disturbances, physiological mechanisms remain stable, thereby maintaining homeostasis (Habibu et al. 2019). The observed time-of-day effect on physiological parameters likely reflects circadian rhythms and daily environmental fluctuations, influencing thermal regulation independently of diet. These findings are consistent with studies demonstrating that RR and RT are reliable indicators of heat stress but are primarily influenced by ambient temperature rather than diet composition, even in diets with low forage fiber content (Berihulay et al. 2019), as illustrated in Fig. 2.

Fat deposition occurs when energy intake exceeds the animal’s requirements (Robelin 1986). Therefore, the lower ADG observed in goats fed WC likely reflects feed sorting behavior, which reduced DMI and consequently ME intake (NRC 2007), resulting in lower BW at slaughter. However, because the animals were slaughtered at a relatively young age, no effects were observed on hot carcass weight or hot carcass yield.

Leg tissue composition is commonly evaluated as a representative measure of carcass composition because it reduces time, cost, and waste compared with whole-carcass dissection (Silva Sobrinho et al. 2011). In the present study, neither the CON diet nor non-forage diets affected the proportions of muscle, bone, fat, or other tissues in the leg. The absence of dietary effects on fat proportion in the leg supports the carcass results.

In ruminants, fat deposition typically begins in visceral depots, followed by intermuscular, subcutaneous, and finally intramuscular depots (Schumacher et al. 2022). Goats, in particular, tend to prioritize fat accumulation in abdominal and visceral depots (Yalcintan et al. 2024), which serve as energy reserves during periods of feed scarcity (Mtenga et al. 2003). For this reason, SFT alone may not be a reliable indicator of total fat deposition in growing goats (Yalcintan et al. 2024).

Greater energy intake in animals fed the CON diet might be expected to increase kidney fat deposition. However, because kidney fat deposition is associated with visceral fat accumulation and may negatively affect carcass yield (Liu et al. 2024), factors influencing ruminal fermentation patterns may also contribute to fat dynamics. High-starch diets are rapidly fermented in the rumen and may disrupt microbial balance, whereas, whole grains ferment more slowly, contributing to greater ruminal stability throughout the day (Carlis et al. 2021). Recent studies (Zhang et al. 2021, 2024) have demonstrated associations between rumen microbiota composition and fat deposition in sheep, which may partially explain the patterns observed in the present study.

Overall, DMI and, consequently, CP and energy intake were key determinants of animal performance. Although the WC diet showed higher DM digestibility, the substantial reduction in DMI decreased the total supply of metabolizable nutrients. Thus, despite the improved digestive efficiency, these results suggest a reduction in total nutrient absorption. The increase in DM and EE digestibility was insufficient to compensate for this reduction in absolute terms, resulting in lower availability of energy and protein for growth. In addition, the reduced CP intake likely limited nitrogen supply to ruminal microorganisms, thereby reducing microbial protein synthesis and the supply of metabolizable protein.

The importance of fiber in maintaining ruminal health is well established. Although ruminal fermentation parameters were not evaluated, limiting a more detailed interpretation, the lower NDF intake observed in the WC diet reduced rumination time and may have affected salivary buffering capacity and ruminal pH stability. However, no clinical signs of acidosis were observed, consistent with previous reports.

## Conclusion

WM can replace WC in non-forage diets, while maintaining satisfactory animal performance, carcass traits, and leg tissue composition without promoting the feed sorting behavior observed in goat kids fed the WC diet.

## Data Availability

The data that supports the findings of this study are available from the corresponding author upon reasonable request.
